# Cardiac surgery for a right atrial myxoma with traumatic intracranial hemorrhage: a case report

**DOI:** 10.1186/s13019-023-02402-2

**Published:** 2023-10-17

**Authors:** Maki Ichinose, Masanori Ogiwara, Masahiko Ozaki, Yoshifumi Nishino, Kensuke Tanaka

**Affiliations:** 1 Department of Anesthesiology, Showa General Hospital, Tokyo, Japan; 2 Division of Cardiovascular Surgery, Showa General Hospital, Tokyo, Japan

**Keywords:** Brain computed tomography, Cardiac surgery, Cardiopulmonary bypass, Intracranial hemorrhage, Right atrial myxoma

## Abstract

**Background:**

The timing of cardiac surgery with cardiopulmonary bypass (CPB) for intracranial hemorrhage is controversial.

**Case presentation:**

We report the case of an 82-year-old woman who was transferred to our hospital because of a head injury. Brain computed tomography (CT) revealed traumatic intracranial hemorrhage, and transthoracic echocardiography revealed a giant right atrial myxoma. After confirming the disappearance of intracranial hemorrhage on brain CT, cardiac surgery with CPB was performed, which was uneventful.

**Conclusions:**

For an uneventful surgery, the optimal timing of cardiac surgery with CPB in patients with giant right atrial myxoma and intracranial hemorrhage should be based on brain CT.

## Background

Cardiac myxomas are intracardiac lesions that account for 50% of benign primary cardiac tumors [1]. Cardiac myxomas rarely occur in the right atrium (RA; 10–20%) [1]. They usually require immediate surgical excision to prevent embolic events and sudden cardiac death [2]. However, surgery may sometimes be difficult because of the patient’s condition. We present the case of a woman with a giant right atrial myxoma and traumatic subarachnoid and subdural hemorrhage who underwent cardiac surgery at an appropriate time and achieved a good outcome.

## Case presentation

**Fig. 1:**
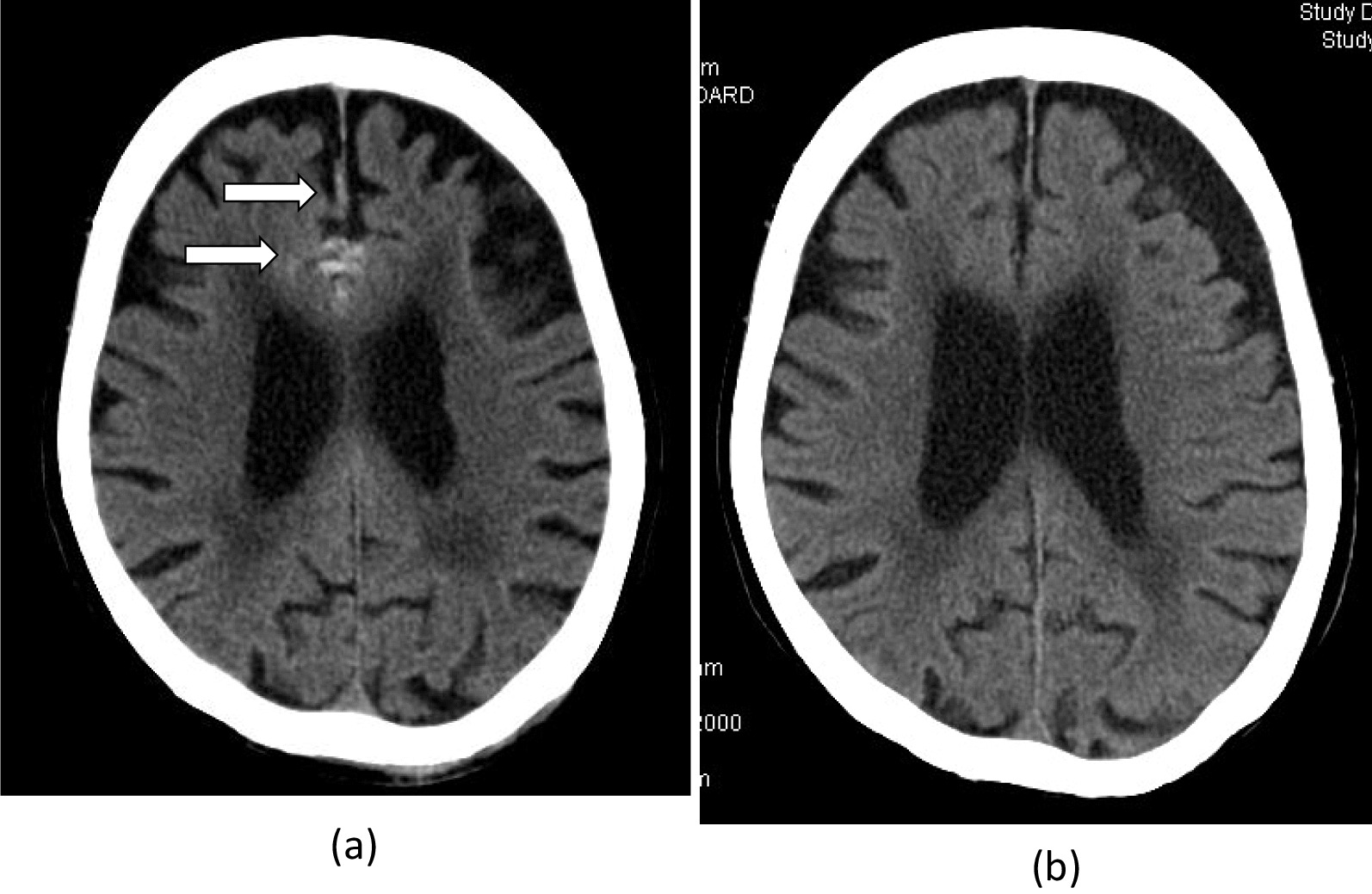
**a** Brain computed tomography (CT) on admission showing subarachnoid and subdural hemorrhages (arrows). **b** Repeat brain CT showing the disappearance of the subdural hemorrhage

**Fig. 2:**
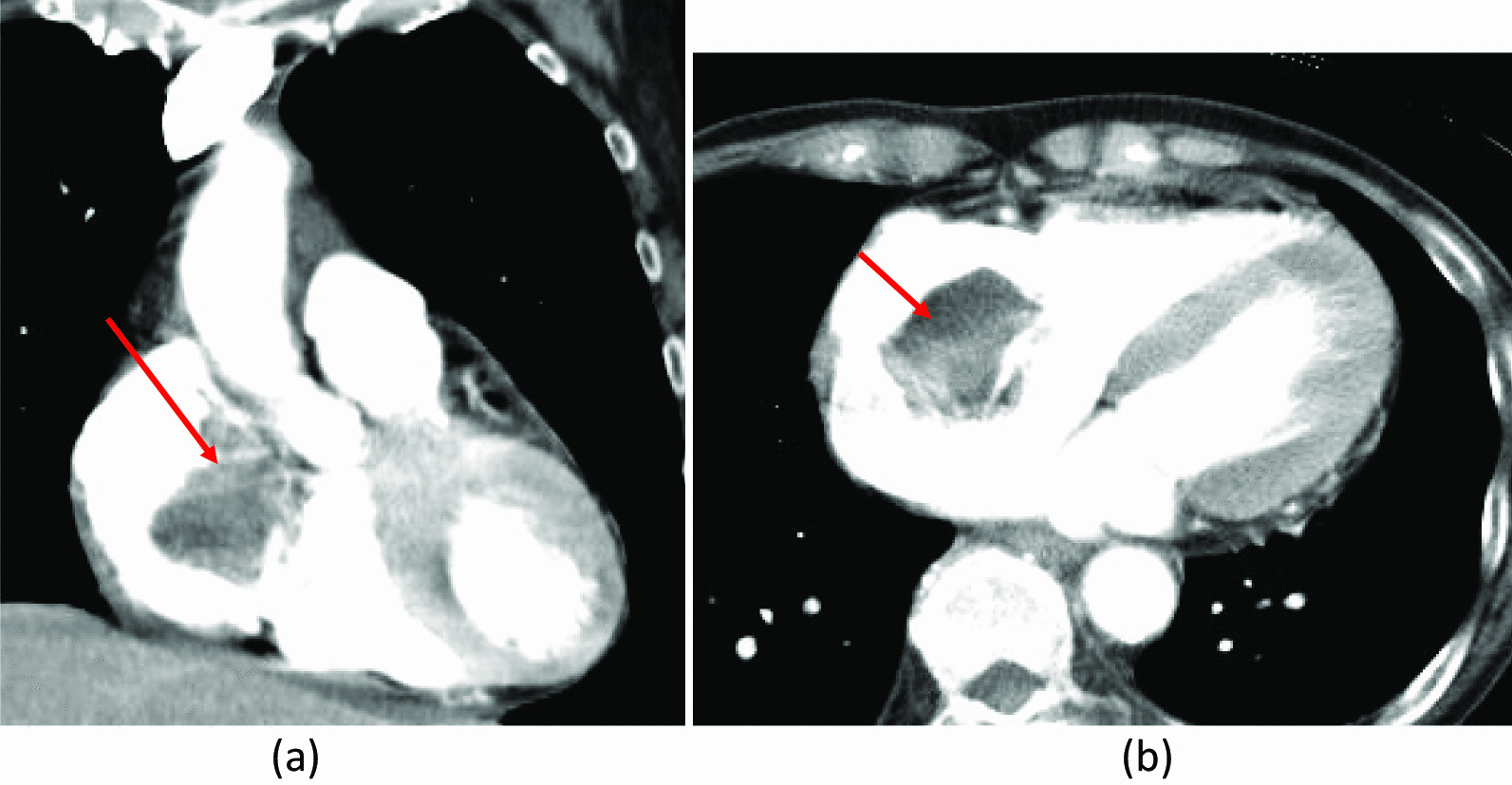
Enhanced computed tomography revealing a large defect in the right atrium (arrows). **a** Coronal view. **b** Axial view

**Fig. 3:**
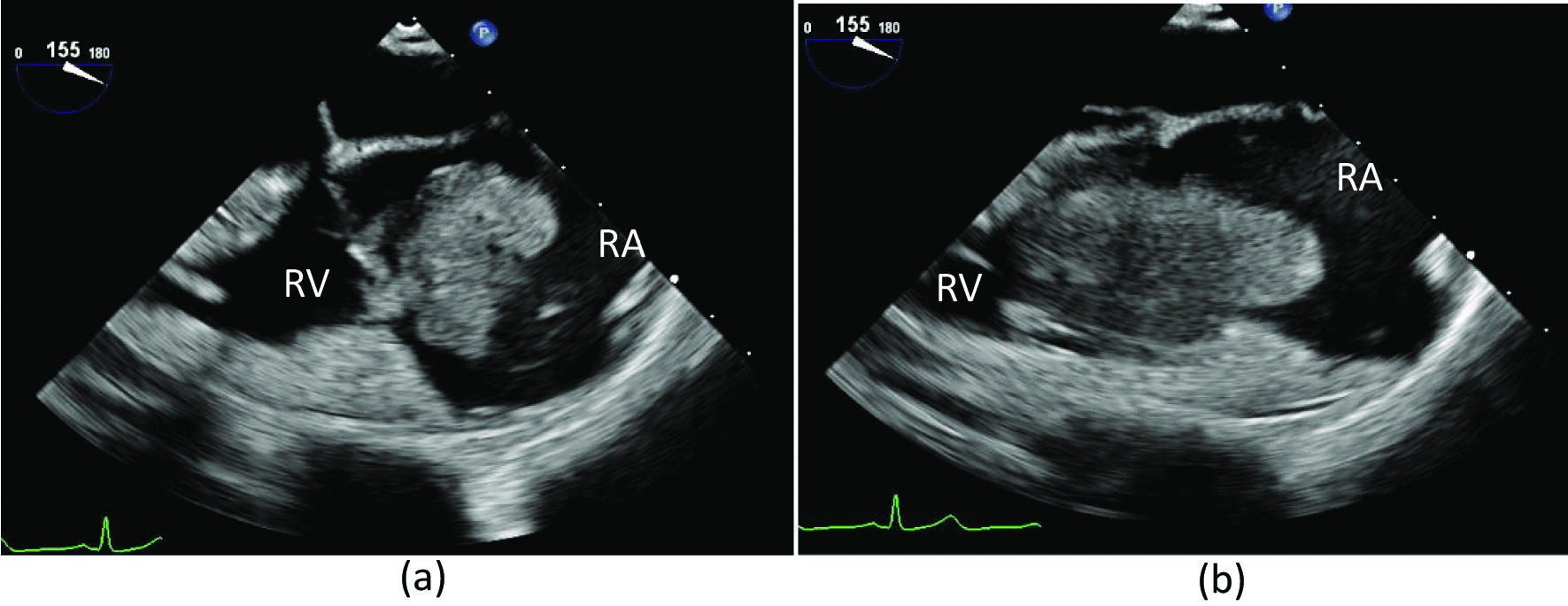
**a** Transesophageal echocardiography showing a large mass with heterogeneous echogenicity. **b** The mass passed through the tricuspid valve to the right ventricle in the diastolic phase

An 82-year-old woman was hospitalized at our emergency center due to a head injury after syncope. She was on anticoagulant therapy due to a previous cerebral infarction. Upon arrival at the emergency medical center, her consciousness was clear, and other vital signs were also stable (heart rate, 74 bpm/min; blood pressure, 109/45 mmHg; respiratory rate, 22 breaths/min; oxygen saturation, 95% [room air]). Signs of a head injury were only found on physical examination. The laboratory data were almost normal except for C-reactive protein (7.91 mg/dL), platelets (9.9 × 10^4^L), D-dimer (234 μg/ml), and activated partial thromboplastin time (APTT) (42.5 s). Electrocardiogram showed regular sinus rhythm. Brain computed tomography (CT) revealed traumatic subarachnoid hemorrhage (SAH) and subdural hemorrhage (Fig. 1a). Full body enhanced CT revealed a large defect in the right atrium (Fig. 2) and a small deep venous thrombus, but no pulmonary embolism. Neither mass lesions nor enlarged lymph nodes were observed on enhanced CT. Consequently, we performed transthoracic echocardiography (TTE), which showed a large, highly echogenic, mobile mass in the right atrium (55 × 45 mm) connected to the anterior free wall and prolapsing through the tricuspid valve into the right ventricle during diastole. Doppler echocardiography revealed a peak pressure gradient of 36 mmHg between the right atrium and ventricle. Color Doppler revealed a moderate tricuspid valve regurgitation (TR), moderate mitral valve regurgitation, and moderate aortic valve regurgitation. The tumor seemed attached to the tricuspid valve, and we could not measure it. The wall motion of the left ventricle was normal, no asynergy was observed, and ejection fraction was 66%. The wall motion of the right ventricle was also normal (Tricuspid annular plane systolic excursion, 20 mm; Systolic tricuspid annulus migration velocity, 11 cm/s). As the tumor could be a thrombus, she was started on unfractionated heparin 10000U per day (body weight 42 kg), and APTT was controlled at > 55 s. The following day, the D-dimer level had decreased to 47.5 μg/ml, but the size of the tumor remained unchanged. Based on the above, we established the diagnosis of a right atrial myxoma with intracranial hemorrhage. Her vital signs were stable, and due to cerebral hemorrhage, immediate surgical excision of the right atrial mass could not be performed. We reperformed physical examination, TTE, laboratory tests, and brain CT several times following her admission. The waiting period was uneventful. On the 15th day, brain CT showed no signs of SAH and only a slight subdural hemorrhage (Fig. 1b). On the 18th day, cardiac surgery with cardiopulmonary bypass (CPB) was performed. Transesophageal echocardiography (TEE) confirmed the TTE finding (Fig. 3a and b). The myxoma appeared as if it had broken into pieces and had been embolized to the pulmonary arteries.

**Fig. 4:**
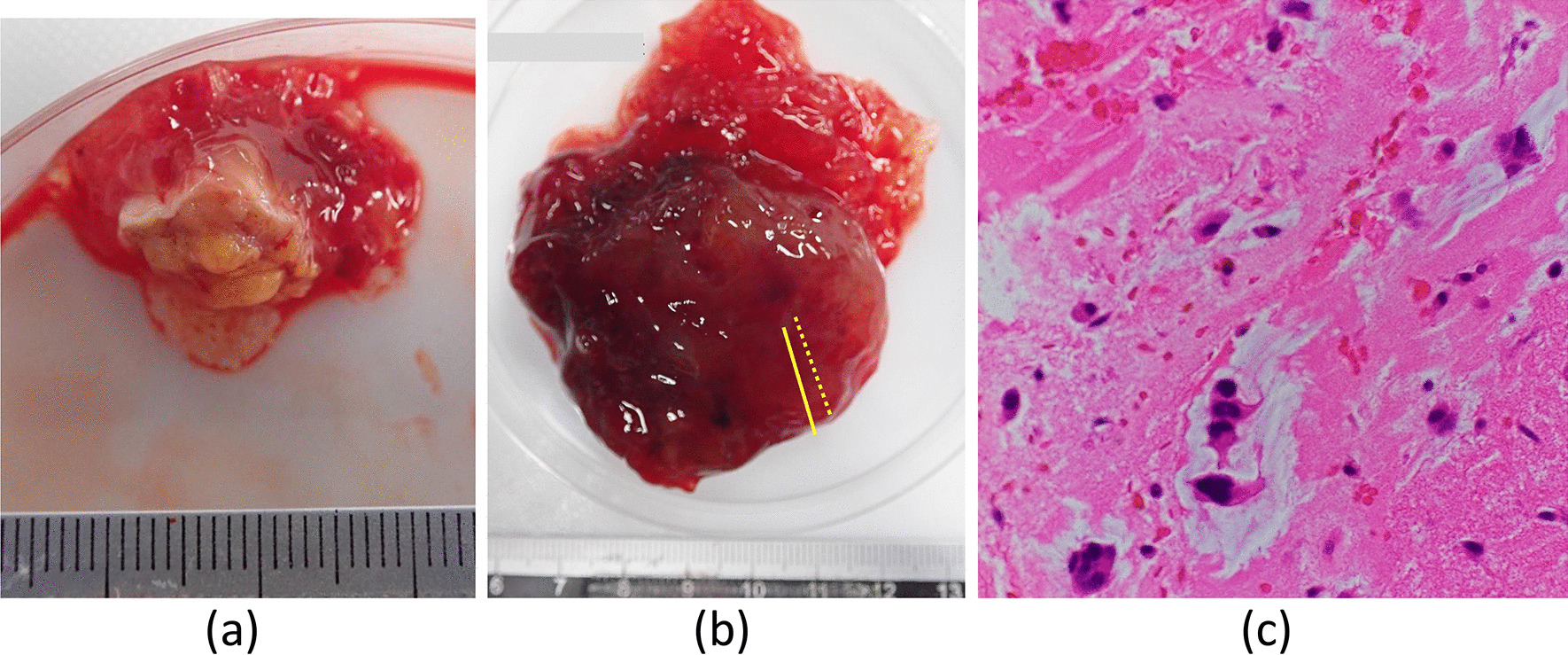
**a** One part of the excised tumor comprising a 25 × 15 × 15 mm mass and a 12 × 11 × 6 mm section of the right atrial wall. **b** The other part comprising a 92 × 50 × 30 mm mass. **c** The chromatin histology sections cut between the solid and dotted lines in (**b**) showing spindle-shaped and star-shaped cells

The surgical approach involved a median sternotomy. CPB was established between the ascending aorta and the supra vena cava (SVC) via the SVC and infra vena cava via the femoral vein. A normal dose of unfractionated heparin (300 units/kg) was administered. During CPB, the activated clotting time was maintained at approximately 400 s. The right atrium was opened, and a large red mass was found attached to the anterior wall of the atrium. The mass was excised and was found to comprise two parts: a small mass that ran along the atrial wall to which the tumor stalk was attached (Fig. 4a) and a large detached mass (Fig. 4b). The defect was repaired using an autologous pericardial patch. The tricuspid valve leaflets and subvalvular apparatus seemed normal. Tricuspid annuloplasty was performed using a 30-mm tricuspid annuloplasty ring (Physio Tricuspid annuloplasty ring; Edwards Lifesciences Co., Tokyo, Japan) because TEE showed moderate TR and the tricuspid annulus was dilatated. CPB was slowly discontinued with pacing. The surgery was uneventful.

The giant mass (92 × 50 × 30 mm) was rough and covered in a gelatinous substance. Histopathological examination showed typical myxoma cells interspersed in the mucus matrix (Fig. 4c).

On the 1st postoperative day, the patient was extubated. The brain CT showed no hemorrhage 24 days post-operation. Her consciousness was fine, but physical activity was reduced; thus, she was transferred to a rehabilitation hospital on the 32th day after brain CT, and there were no signs of hemorrhage and infarction.

## Discussion

To the best of our knowledge, there have been no reports of myxoma with intracranial hemorrhage without cerebral infarction. Hence, it was difficult to determine the optimal timing of cardiac surgery in this case.

Cardiac surgery under CPB with complete excision of the myxoma within a few days of the onset of hemorrhage with cardiac infarction has been reported. Kato et al. [3] performed surgical removal of a myxoma within 24 h after the onset of SAH with cerebral infarction and reported a successful outcome. They decided to perform the surgery because the intracranial hemorrhage was very small [3]*.* Kano et al. [4] performed surgery on patients with infective endocarditis with large cerebral infarction and hemorrhage 2 days after the cerebral infarction occurred and reported a good outcome.

Otherwise, the Japanese Circulation Society 2017 Guideline on Prevention and Treatment of Infective Endocarditis recommends that in patients with infective endocarditis, surgery should be postponed for at least 4 weeks after a new intracranial hemorrhage is detected [5]. However, waiting 4 weeks is not always practical because the condition of the patient may worsen. Okita et al. [6] reported on the timing of surgery for active infective endocarditis with cerebral complications. They showed that the risk of complications was lower when surgery was performed 1 week after the onset of intracranial hemorrhage than when it was performed within 1 week of the onset. In addition, waiting at least 3 weeks may be sufficient to decrease the overall risk in these circumstances.

In the case reported here, immediate surgical excision was required, but the risk of brain complications was high. There was a risk of pulmonary embolism, but she did not have cerebral infarction at that time. Thus, according to the guideline, elective cardiac surgery was selected. Our case had a similar hemorrhage size to that reported by Kato et al. [3], but our patient had only cerebral hemorrhage without cerebral infarction. Thus, we waited until cerebral hemorrhage disappeared. We evaluated the patient’s general condition. In addition, we checked the size of the myxoma and tricuspid regurgitation and stenosis using TTE. We also performed a brain CT to determine the size of the SAH and subdural hemorrhage 6, 12, and 24 h and a few days after the first detection of hemorrhage according to the general course of traumatic intracranial hemorrhage. On the 15th day after admission, the intracranial hemorrhage disappeared, and no new intracranial event was found on brain CT. Hence, cardiac surgery using a normal amount of unfractionated heparin, with excision of the right atrial mass, was performed. Postoperative brain CT showed no other brain complications.

### Limitations

An important limitation of this study is that the hemorrhage was small, which meant that we might have been able to perform surgery within 4 weeks and still have a good outcome.

## Conclusions

A right atrial myxoma with traumatic intracranial hemorrhage is rare. Moreover, the timing of cardiac surgery with CPB is controversial. Here, we demonstrated the use of brain CT to determine the optimal timing for an uneventful surgery.

## Data Availability

The data used to support the findings of this study are restricted by the ethics board of Showa General Hospital to protect patient privacy. Data may be provided for researchers who meet the institutional criteria to obtain access to confidential data.

## Abbreviations

APTT: Activated partial thromboplastin time
CPB: Cardiopulmonary bypass
CT: Computed tomography
LA: Left atrium
LV: Left ventricle
RA: Right atrium
RV: Right ventricle
SAH: Subarachnoid hemorrhage
SVC: Supra vena cava
TEE: Transesophageal echocardiography
TTE: Transthoracic echocardiography
TR: Tricuspid valve regurgitation
